# Stable GaSe-Like Phosphorus Carbide Monolayer with Tunable Electronic and Optical Properties from Ab Initio Calculations

**DOI:** 10.3390/ma11101937

**Published:** 2018-10-11

**Authors:** Xiaolin Cai, Zhili Zhu, Weiyang Yu, Chunyao Niu, Jianjun Wang, Baoji Wang, Xiaohua Li, Liwei Zhang, Ruiqi Zhao, Yu Jia

**Affiliations:** 1 School of Physics and Electronic Information Engineering, Henan Polytechnic University, Jiaozuo 454000, China; caixiaolin@hpu.edu.cn (X.C.); yuweiyang@hpu.edu.cn (W.Y.); wbj@hpu.edu.cn (B.W.); lixiaohua@hpu.edu.cn (X.L.); lwzhang@hpu.edu.cn (L.Z.); 2 Key Laboratory for Special Functional Materials of Ministry of Education, School of Physics and Electronics, Henan University, Kaifeng 475004, China; 3 International Laboratory for Quantum Functional Materials of Henan and School of Physics and Engineering, Zhengzhou University, Zhengzhou 450001, China; zlzhu@zzu.edu.cn (Z.Z.); niuchunyao@zzu.edu.cn (C.N.); 4 College of Science, Zhongyuan University of Technology, Zhengzhou 450007, China; hnxhwjj@163.com; 5 School of Materials Science and Engineering, Henan Polytechnic University, Jiaozuo 454000, China

**Keywords:** density functional theory, GaSe-like structure, phosphorus carbide monolayer, electronic structures, optical properties, vacancy, magnetism

## Abstract

On the basis of density functional theory (DFT) calculations, we propose a stable two-dimensional (2D) monolayer phosphorus carbide (PC) with a GaSe-like structure, which has intriguing electronic and optical properties. Our calculated results show that this 2D monolayer structure is more stable than the other allotropes predicted by Tománek et al. [*Nano Lett., 2016, 16, 3247–3252*]. More importantly, this structure exhibits superb optical absorption, which can be mainly attributed to its direct band gap of 2.65 eV. The band edge alignments indicate that the 2D PC monolayer structure can be a promising candidate for photocatalytic water splitting. Furthermore, we found that strain is an effective method used to tune the electronic structures varying from direct to indirect band-gap semiconductor or even to metal. In addition, the introduction of one carbon vacancy in such a 2D PC structure can induce a magnetic moment of 1.22 µ_B_. Our findings add a new member to the 2D material family and provide a promising candidate for optoelectronic devices in the future.

## 1. Introduction

Research in two-dimensional (2D) materials, as initiated by the successful exfoliation of graphene, has experienced an explosive growth in recent years owing to their unique properties and promising applications in many fields. So far, scientists have realized the synthesis of 2D materials composed of one type of elements in main groups III (borophene [1,2]), group IV (graphene [3], silicene [4], germanene [5], and stanene [6]), group V (phosphorene [7], arsenene, and antimonene [8,9]), and group VI (tellurene [10,11]). Besides these one-element 2D materials, the MX counterparts composed of III–V and III–VI elements (M = B, Ga, and In; X = N, S, Se, and Te) have also been widely investigated as a result of their novel electronic, mechanical, and optical properties [12,13,14,15,16,17]. Hexagonal boron nitride (*h*–BN) presents excellent insulation, extremely high thermal and chemical stabilities, and has been used as a coating and a nanolubricant in extreme environments [18,19]. The ultimate dimension reduction of 3D III–VI layered materials leads to the 2D monolayers possessing novel electronic and optical properties [17]. Furthermore, monolayer *h*–BN, GaS, GaSe, and InSe sheets have been obtained in experiments [13,14,20,21]. Inspired by the superior characteristics of these binary monolayers, it is essential to explore other 2D counterparts and reveal their intrinsic properties. Very recently, using a topology-scaling algorithm, Ashton et al. found 826 stable layered materials that are considered as candidates for forming 2D monolayers via exfoliation [22]. Subsequently, Mounet et al. identified 1825 compounds that are either easily exfoliated or potentially exfoliated into a single atomic layer by high-throughput computation [23]. In addition to these experimental materials which are likely to be exfoliated into monolayers, it is also of great significance to find 2D materials with no layered counterparts or layered materials that are likely to be successfully prepared experimentally in the future.

It is well known that graphene and phosphorene are composed of the C element in group IV and the P element in group V, respectively. Graphene possesses extremely high mobilities, while its intrinsic dispersion is gapless, resulting in a relatively large ‘off’ current and thus a low ‘on-off’ ratio [24,25,26,27]. Although phosphorene has both high carrier mobilities and a suitably fundamental band gap, the quality degradation under atmospheric conditions impedes its applications [28,29]. Therefore, it is intriguing to find a stable 2D binary counterpart composed of “C” and “P” elements, and more importantly, such a compound which exhibits properties superior to graphene and phosphorene. Tománek et al. have proposed six stable 2D allotropes of phosphorus carbide (PC) that can be metallic or semimetallic with an anisotropic Dirac cone or direct gap semiconductors with tunable gap by inner-layer strain [30]. Zheng et al. examined the stabilities and electronic properties of various PC structures and found that the 3D layered PC with GaSe-like structure is the most energetically stable one [31]. To our knowledge, there is still a lack of literature exploring the stability, and electronic and optical properties of the monolayer PC with GaSe-like structure.

In this paper, we investigate the crystal structure, stability, and electronic properties of GaSe-like monolayer PC by density functional theory (DFT) calculations. The calculated results show that this monolayer is a semiconductor with a direct-gap of 2.65 eV, which thus results in excellent optical absorption. The electronic structure exhibits a sensitive response to strain, inducing a transition from direct to indirect semiconductors or even to metal. More intriguingly, the carbon vacancies can induce obvious magnetism in such a monolayer PC. Our new findings add a potential member to the 2D material family and provide a promising candidate for optoelectronic devices in the future.

## 2. Materials and Methods

Our simulations are performed based on DFT with the vienna ab initio simulation package (VASP) [32,33]. When we deal with the exchange correlation energy and core electrons in solids and surfaces, there are many functionals available, such as the generalized gradient approximation (GGA) by the perdew–burke–ernzerhof (PBE) [34] functional, the semiclassical neutral atom theory [35], the kinetic-energy-density dependent semilocal exchange-correlation functionals [36], and the revised PBE-GGA improving equilibrium properties [37]. For the simple PC structure, we can get accurate results by using the standard PBE-GGA functional with the van der Waals (vdW) corrections [38,39], and the projector augmented wave (PAW) pseudopotentials [40,41]. A vacuum of 20 Å is used to isolate interactions between adjacent supercells. We have also checked the results using a larger vacuum of 30 Å. A kinetic energy cutoff of 500 eV is employed for the plane wave basis set. All the atoms in the structure are fully relaxed until the total energy and the Hellmann–Feynman force on each atom are less than 10^−5^ eV and 0.01 eV/Å, respectively. The phonon spectrum calculation is performed with an approach implemented in the Phonopy code [42]. A supercell of 6 × 6 × 1 is used for the ab initio molecular dynamics (AIMD) simulation based on a canonical ensemble (NVT) [43]. The band gap structures are checked by the hybrid Heyd–Scuseria–Ernzerhof (HSE06) exchange-correlation functional [44].

To examine the stability of the GaSe-like 2D PC, the cohesive energy is calculated by the formula, *E*_c_ = (*E*_total_ − 2*E*_C_ − 2*E*_P_)/4, here *E*_c_ is the cohesive energy of per “average” atom with respect to the isolated atom, *E*_total_ is the total energy of monolayer PC unit cell, and *E*_C_ and *E*_P_ are the energies of the isolated C and P atoms, respectively. On the basis of the linear response theory (DFT), the optical absorption coefficient *I*(*ω*) is calculated by the formula $I\left(\omega \right)=\sqrt{2}\omega {\left[\sqrt{\varepsilon_{1}{\left(\omega \right)}^{2}+\varepsilon_{2}{\left(\omega \right)}^{2}}-\varepsilon_{1}\left(\omega \right)\right]}^{1/2}$, where $\omega ,\;\varepsilon_{1}$ and $\varepsilon_{2}$ are the frequency of light, and the real and imaginary parts of the complex dielectric function, respectively. In order to ensure the accuracy of the results, we use the HSE functional to calculate the absorption coefficient.

## 3. Results

### 3.1. Crystal Structure and Stability

**Figure 1:**
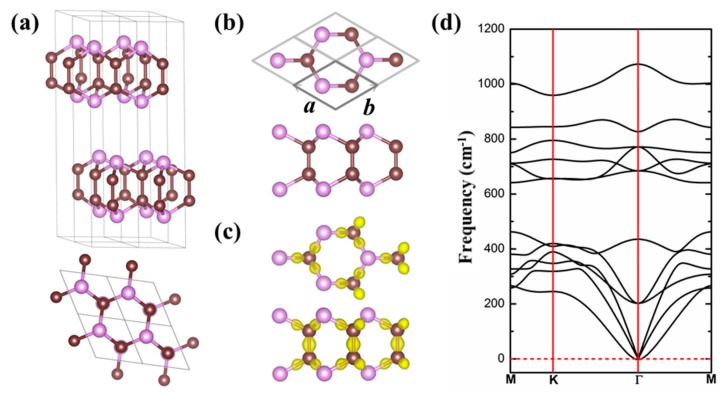
(**a**,**b**) Are the top and side views of the crystal structures of GaSe-like bulk and 2D monolayer phosphorus carbide (PC), respectively. Panel (**c**) is the charge density difference of GaSe-like monolayer PC. Panel (**d**) is phonon spectrum of 2D PC monolayer. The purple and brown balls represent P and C atoms, respectively. In panel (**c**), the isosurface value is 0.03 *e*/$a_{0}^{3}$.

Firstly, we consider the crystal structure and electronic properties of GaSe-like bulk PC with space group *P*63/*mmc* (194). This bulk PC has an obvious layered structure, in which the intralayer consists of four sublayers stacked by the covalent bonds in the sequence P–C–C–P, and the interlayers are held together by the weak vdW forces, shown in Figure 1a. The relevant parameters are presented in Table 1. The calculated lattice constants are *a* = *b* = 2.88 Å and *c* = 13.83 Å, agreeing well with the result reported by Zhuang et al. [17], which indicates the reliability of our computations. The C–C and C–P bond lengths are ~1.55 Å and ~1.88 Å, respectively. The P–C–P (C–P–C) and P–C–C bond angles are 100.31° and 117.55°, respectively. The cohesive energy is 5.50 eV/atom, and the charge transfer from P to C atom is 1.09 *e*. The calculated result using the PBE scheme shows that the GaSe-like bulk PC is a semiconductor with an indirect band gap of 1.38 eV (2.33 eV by HSE06), and the conduction band minimum (CBM) is at the K point in the Brillioun zone while the valence band maximum (VBM) is near K point, shown in Figure S1.

We next study the properties of GaSe-like monolayer PC. Figure 1b presents the optimized crystal structure of the PC monolayer. The structural parameters, cohesive energies, and charge transfer of 2D PC are listed in Table 1, slightly different from those of the bulk PC structure. The cohesive energy of GaSe-like PC is 5.46 eV/atom. Using the same computational method, we evaluate the cohesive energy of the most stable monolayer α_2_-PC predicted by Tománek et al. [30] as 5.03 eV/atom, 0.43 eV/atom smaller than that of our proposed monolayer PC. This result indicates a higher stability of such a 2D GaSe-like PC in energy. Furthermore, the charge difference distribution clearly shows the charge transfer from P to C (Figure 1c), which results from the different electronegativity of C (2.55) and P (2.19). The Bader charge analysis reveals that the charge transfer from P to C is 1.11 *e*. It was noticed that the larger charge transfer may enhance the stability of the 2D PC monolayer. In the periodic table, the C element is on the left of P, and the compound of C and P should traditionally be called ‘carbon phosphide’ [45,46,47]. For our proposed C and P compound, the C atom receives electrons while the P atom loses them. From the point of view of gain and loss of electrons, the C and P compound in this paper is termed ‘phosphorus carbide (PC)’, in line with Tománek et al. [30].

To further examine the thermodynamic stability of GaSe-like 2D PC, we calculated the phonon spectra, as displayed in Figure 1d. We can clearly observe no imaginary frequencies in the phonon spectra, revealing that the 2D PC is thermodynamically stable. We also performed AIMD simulations within 6 *ps* at 300 K. In the whole AIMD simulation, the honeycomb structure is well maintained and the total energy oscillates within a small range (Figure S2), indicating that the structure is dynamically stable at room temperature. We further evaluated its mechanical stability by calculating its elastic constants. The two independent elastic constants *C*_11_ and *C*_12_ are 284 N m^−1^ and 23 N m^−1^, respectively, which satisfy the Born criteria for mechanical stability of hexagonal structures [48].

### 3.2. Electronic and Optical Properties

**Figure 2:**
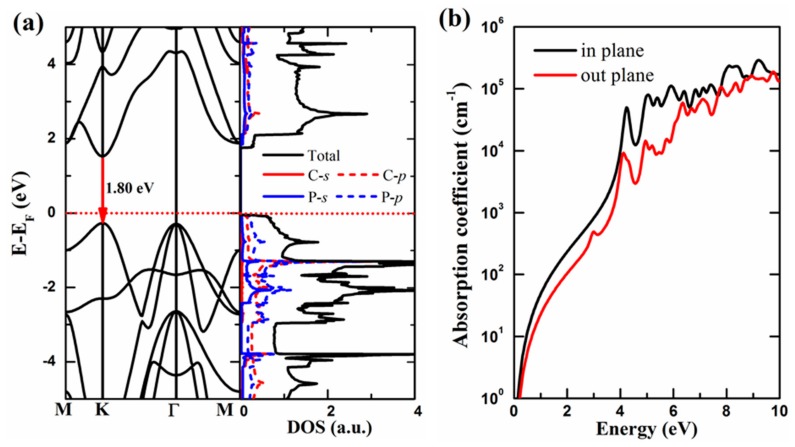
(**a**) Electronic structures (band structures and projected density of states (PDOS)), and (**b**) optical absorption coefficients of the GaSe-like 2D PC. In panel (**a**), the Fermi level is set to zero.

In the following section, we will discuss the electronic properties of the GaSe-like PC monolayer. Figure 2a shows the band structure and the projected density of states (PDOS) of the 2D PC obtained from the PBE functional. We found that 2D PC monolayer exhibits semiconductor properties with a direct band-gap of 1.80 eV. The VBM and CBM, which mainly arise from the *p* orbitals of C and P atoms, are both located at the K point. The PDOS of C and P atoms overlap in the same energy levels, indicating the bond formation between C and P atoms. The bond formation can also be confirmed by the charge difference distribution shown in Figure 1c.

We examine the variation of the electronic band structure of PC upon increasing the number of layers. Like most 2D materials, we find that the PC monolayer also shows thickness-dependent electronic properties (see Figure S3). With the increase in the number of layers, the multilayer PC transforms to an indirect band-gap semiconductor due to the VBM shifting to the Γ point (Figure S3a), and its band structure will approach that of bulk PC because the VBM again shifts to near the K point at a certain number of layers. Meanwhile, both the band gap and the average energy per atom of the PC multilayer decrease, and gradually approach those of GaSe-like bulk PC (see Figure S3b,c).

The PBE scheme usually underestimates the band-gap values of semiconductors. Using the more accurate HSE06 [44] functional, the accurate band gap of GaSe-like 2D PC is 2.65 eV, which is indeed larger than those of α_0_-, α_1_-, and β_1_-PC reported by Tománek et al. [30]. The value of the band gap suggests that the proposed 2D PC monolayer can be a promising wide-band-gap semiconductor. Furthermore, it is interesting to explore the potential application of such 2D material in photocatalytic water splitting. To be an excellent photocatalyst for water splitting, two criteria should be met: firstly, the material must exhibit the property of high optical absorptions; secondly, its band edges of the VBM and CBM must cover the redox potentials of water effectively [49]. To meet this, firstly, we calculate the optical absorptions of the GaSe-like PC monolayer (Figure 2b), which shows that both the in-plane and the out-of-plane optical absorptions are as large as up to 10^5^ cm^−1^. Secondly, we determine the band edges of the GaSe-like PC monolayer relative to the vacuum level using the HSE functional. The calculated CBM and VBM are −4.00 and −6.69 eV, respectively, which, like MoS_2_, entirely straddle the redox potentials of water [42], i.e., −4.44 eV (reduction potential for H^+^/H_2_) and −5.67 eV (oxidation potential for O_2_/H_2_O), respectively. Both the optical absorption and band edge alignments indicate that our proposed GaSe-like 2D PC monolayer can be a promising candidate for photocatalytic water splitting.

### 3.3. Tunable Electronic Property by Strain

**Figure 3:**
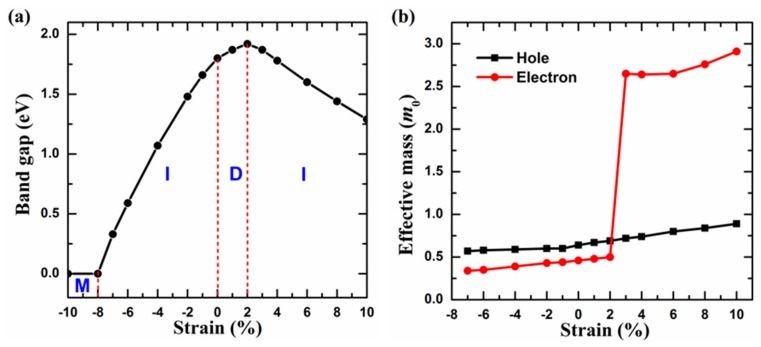
Strain effects on (**a**) the band gaps, and (**b**) the effective masses of 2D PC. In panel (**a**), M, I, and D represent the metal, indirect semiconductor, and direct semiconductor, respectively.

Strain is unavoidable in 2D materials and has been confirmed to be an effective means of tuning the electronic properties. In the following part, we investigate the response of the band gap to the biaxial strain varying from −10% to +10% (Figure 3a), where the plus and minus signs represent the tensile and compressive strains, respectively. It is found that the electronic properties depend precisely on the applied strain. Once the compressive strain is applied, the VBM shifts from the K point to the Γ point, and the system changes from a direct band-gap semiconductor to an indirect one. In addition, with the compressive strain increasing, the band gap decreases until the system becomes a metal under the strain of −8% (see Figure S4). As the tensile strain increases from zero to +2%, the 2D PC monolayer retains the characteristics of a direct band-gap semiconductor, whose band gap increases from 1.80 to 1.92 eV. Furthermore, with the increase of the tensile strain from 2% to 10%, the 2D PC monolayer transforms into an indirect band-gap semiconductor due to the VBM shifting to the M point, which is accompanied by the continuous decrease of the band gap from 1.92 to 1.29 eV. 

Effective mass is an important parameter affecting the carrier mobility of 2D materials. For the GaSe-like 2D PC monolayer, we further determine the effective masses of the electron at the CBM and the hole at the VBM as *m*_e_ = 0.46 *m*_0_ and *m*_h_ = 64 *m*_0_, where *m*_0_ is the rest mass of the electron. The effective mass of the electron is smaller than that of the 2H-MoS_2_ monolayer (0.60 *m*_0_) [50], which manifests that the 2D PC may possess a higher electronic mobility than the 2H-MoS_2_ monolayer. Figure 3b presents the variations of effective masses as a function of the biaxial strain varying from −7% to +10%. On the whole, the effective masses of the hole and electron both decrease under the compressive strain, whereas they increase under the tensile strain. From the above discussion, we learn that the compressive strain changes the position of the VBM to the Γ point, and the tensile strain exceeding +2% makes the CBM shifting to the M point (see Figure S4). It is worth mentioned that the change of the VBM position has little effect on the effective mass of the hole, while the change of the CBM position has a great effect on the effective mass of the electron, causing the effective mass of the electron to rise sharply from 0.50 to 2.65 *m*_0_ when the strain increases from +2% to +3%. The underlying reason for this phenomenon is the electronic characteristics. The flatter the band curve at the VBM or the CBM, the larger the effective mass of the carrier. Since the conduction band with the lowest energy is much flatter at the M point than at the K point (see Figure S4), the effective mass of the electron undergoes a huge change (from 0.50 to 2.65 *m*_0_) after the CBM moves from the K point to the M point under the tensile strain from +2% to +3%. Nevertheless, the effective mass of hole undergoes a gentle change when the VBM shifts from the K point to the M point under compressive strain, which is attributed to the similar curvatures of the valence band with the highest energy at the M and K points (see Figure S4). These results imply that the biaxial compressive strain can reduce the effective masses of the 2D PC monolayer, which may enhance its carrier mobility.

### 3.4. Magnetism Induced by C Vacancy

In 2D materials, there commonly exist defects, such as vacancies, impurities, and grain boundaries, which can remarkably affect the properties of the materials. In this work, we only take the simplest point defect as an example and study its influences on the magnetic properties of the GaSe-like 2D PC. The vacancy is formed by removing one C (V_C_) or P (V_P_) atom in a 4 × 4 × 1 supercell. After fully relaxing the systems with the V_C_ and V_P_, we find that the magnetic moments induced by the V_C_ and V_P_ are 1.22 µ_B_ and 0.12 µ_B_, respectively. The weaker magnetism induced by the V_P_ is mainly distributed on its three nearest C atoms, which can be explained by the fact that the V_P_ induces less unsaturated electrons on the nearest neighbor C atoms (see Figure 1b). 

**Figure 4:**
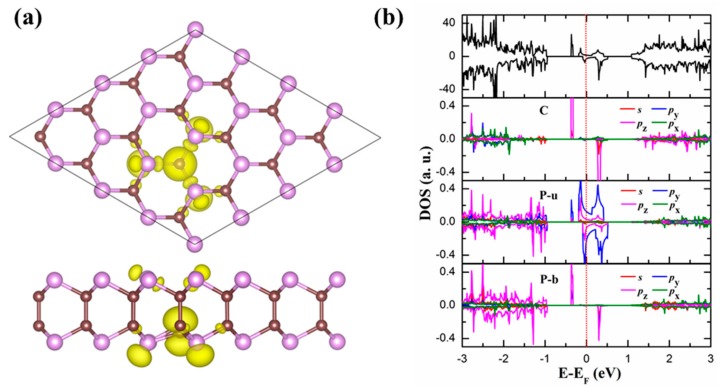
(**a**) Top and side views of the magnetization charge density, and (**b**) total DOS and PDOS of the 2D PC with one C vacancy. In panel (**a**), the isosurface value is 0.003 *e*/$a_{0}^{3}$. In panel (**b**), the Fermi level is set to zero, and C, P-u, and P-b present the nearest C atom, the nearest P atom in the upper sublayer, and the next-nearest P atom in the bottom sublayer.

Here we focus on the magnetic origin of the 2D PC monolayer with the V_C_. Figure 4a presents its spin charge density, which clearly reveals that the spin polarization induced by the V_C_ appears not only at the nearest C atom and the three nearest P atoms, but also at the three next-nearest P atoms in the other sublayer. The magnetism of the nearest C and P atoms can be attributed to the dangling bonds. To further unveil the origin of magnetism on the three next-nearest P atoms in the different sublayer from the V_C_, we calculate the total density of states (DOS) of the 2D PC monolayer with the V_C_ (Figure 4b). It can be clearly seen that the total DOS of spin up and spin down are asymmetric, indicating the existence of magnetism in the system. Additionally, the magnetism originates from the stronger split of *p*_z_ orbitals of the nearest C and the next-nearest P atoms in the other sublayer from the V_C_, and from the weaker split of *p*_y_ and *p*_z_ orbitals of the nearest P atoms. Meanwhile, we also find that there occurs strong *p*_z_ orbital hybridization between the nearest C and next-nearest P atoms, which causes the charge transfer and thus the stronger magnetic properties in the next-nearest P atoms.

## 4. Conclusions

In summary, we report a new 2D GaSe-like PC monolayer with a high stability by means of first-principle calculations within the frame of DFT. Our results show that the 2D PC monolayer exhibits a direct semiconductor with a band gap of 2.65 eV, and thus exhibits superb optical absorptions, and also could be a promising candidate for photocatalytic water splitting. Further studies reveal that applying strain can dramatically tune the electronic structure and effective masses of such a 2D PC monolayer, resulting in a transition from a direct band-gap semiconductor to an indirect one or even a metal. More interestingly, we show that the carbon vacancy can induce remarkable magnetism in such a GaSe-like PC. Our new findings add a new member to the 2D material family, which has a great potential for application in nanodevices.

## Figures and Tables

**Table 1:** Structural parameters of GaSe-like bulk and 2D phosphorus carbide (PC), the cohesive energy *E*_c_, and charge transfer Δ*Q* from P atom to C atoms. *a*, *b*, and *c* represent the lattice constants, *l* is the bond length and *θ* is the bond angle.

Structure	Constants (Å)	*l* (Å)	*θ*	*E*_c_ (eV/atom)	Δ*Q* (*e*)
bulk	*a* = *b* = 2.88 *c* = 13.83	*l*_C-C_ = 1.55 *l*_C-P_ = 1.88	*θ*_PCP_ = 100.31° *θ*_PCC_ = 117.55°	−5.50	1.09
monolayer	*a* = *b* = 2.88	*l*_C-C_ = 1.55 *l*_C-P_ = 1.88	*θ*_PCP_ = 100.27° *θ*_PCC_ = 117.59°	−5.46	1.11

## Supplementary Materials

The following are available online at http://www.mdpi.com/1996-1944/11/10/1937/s1, Figure S1: Band structure of GaSe-like bulk PC, Figure S2: AIMD simulation results of GaSe-like 2D PC monolayer. The upper and bottom illustrations are the initial and final structures of the AIMD simulation, respectively, Figure S3: (a) Electronic band structures of PC bilayer and trilayer, and variations of (b) the band gap and (c) the average energy of PC with thickness (number of PC atomic layers), Figure S4: Band structures of the proposed 2D PC under the biaxial strains.
