# Supplementary material for: Stable GaSe-Like Phosphorus Carbide Monolayer with Tunable Electronic and Optical Properties from Ab Initio Calculations
Source: Materials (Basel). 2018 Oct 11;11(10):1937. doi: 10.3390/ma11101937 (PMC6213067; doi:10.3390/ma11101937)
Supplement: Supplementary file 1 [file materials-11-01937-s001.pdf]

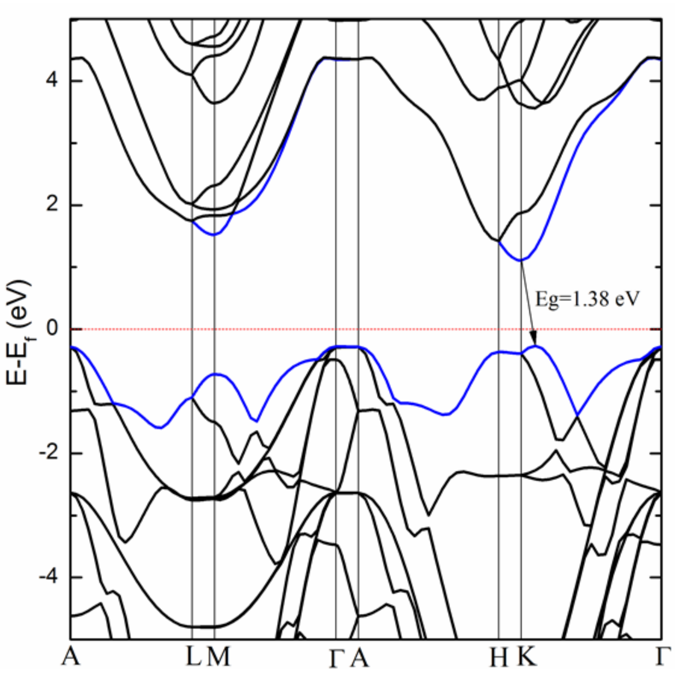

Figure S1. Band structure of GaSe-like bulk PC.

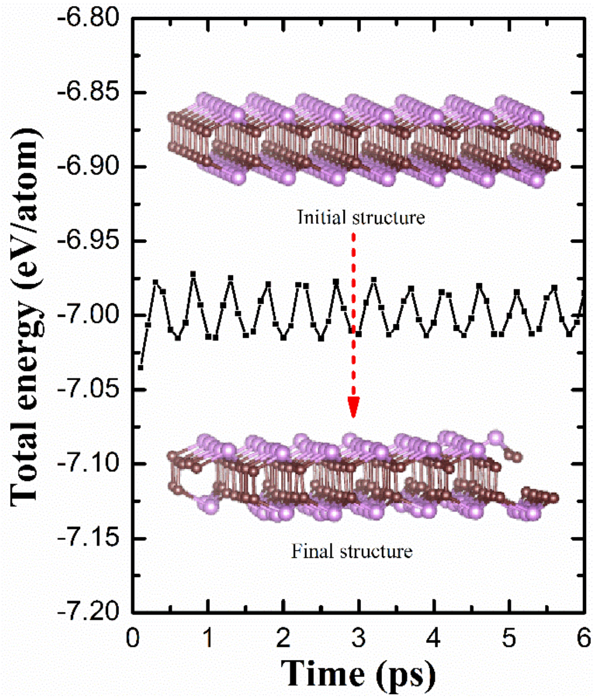

Figure S2. AIMD simulation results of GaSe-like 2D PC monolayer. The upper and bottom illustrations are the initial and final structures of the AIMD simulation, respectively.

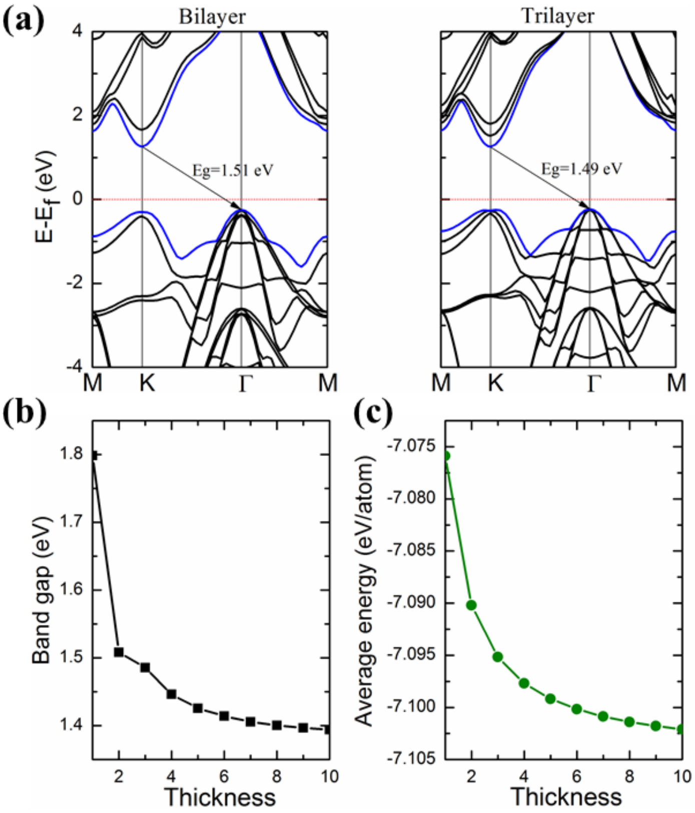

Figure S3. (**a**) Electronic band structures of PC bilayer and trilayer, and variations of (**b**) the band gap and (**c**) the average energy of PC with thickness (number of PC atomic layers).

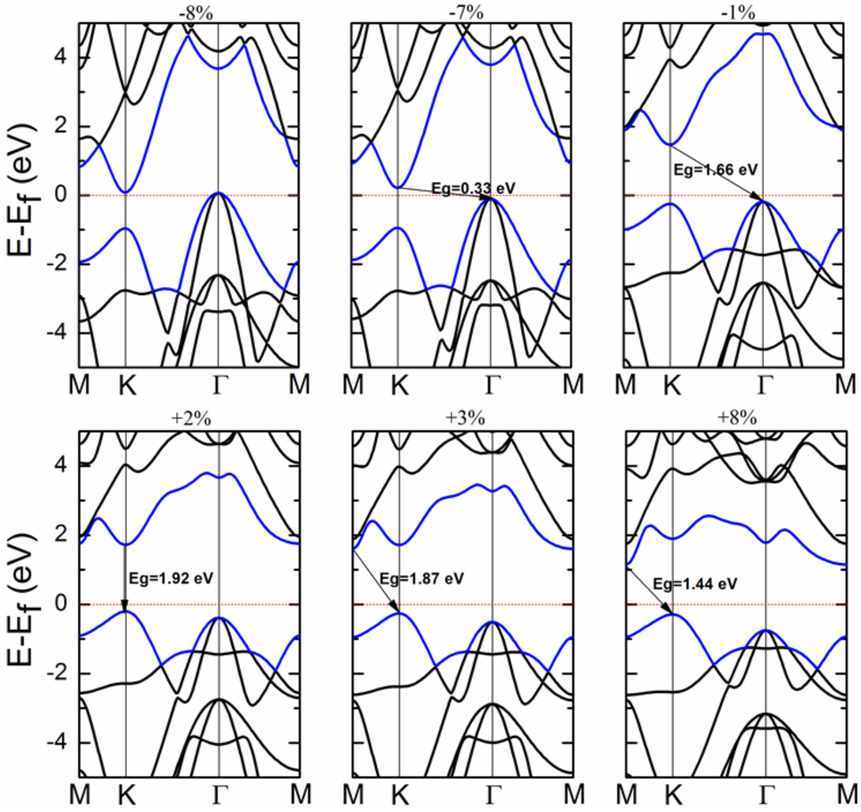

Figure S4. Band structures of the proposed 2D PC under the biaxial strains.
